# Study of Neutron-, Proton-, and Gamma-Irradiated Silicon Detectors Using the Two-Photon Absorption–Transient Current Technique

**DOI:** 10.3390/s24165443

**Published:** 2024-08-22

**Authors:** Sebastian Pape, Marcos Fernández García, Michael Moll, Moritz Wiehe

**Affiliations:** 1 CERN, Esplanade des Particules 1, 1217 Meyrin, Switzerland; marcos.fernandez@cern.ch (M.F.G.); michael.moll@cern.ch (M.M.); m.wiehe@cern.ch (M.W.); 2 Department of Physics-AG Kröninger, TU Dortmund University, 44227 Dortmund, Germany; 3 Instituto de Física de Cantabria (CSIC-UC), Avenida de los Castros, E-39005 Santander, Spain

**Keywords:** solid-state detectors, silicon detectors, device characterisation, radiation damage, two-photon absorption–transient current technique, transient current technique

## Abstract

The Two-Photon Absorption–Transient Current Technique (TPA-TCT) is a device characterisation technique that enables three-dimensional spatial resolution. Laser light in the quadratic absorption regime is employed to generate excess charge carriers only in a small volume around the focal spot. The drift of the excess charge carriers is studied to obtain information about the device under test. Neutron-, proton-, and gamma-irradiated p-type pad silicon detectors up to equivalent fluences of about 7 × 10^15^ $n_{\mathrm{eq}}/cm^{2}$ and a dose of 186 Mrad are investigated to study irradiation-induced effects on the TPA-TCT. Neutron and proton irradiation lead to additional linear absorption, which does not occur in gamma-irradiated detectors. The additional absorption is related to cluster damage, and the absorption scales according to the non-ionising energy loss. The influence of irradiation on the two-photon absorption coefficient is investigated, as well as potential laser beam depletion by the irradiation-induced linear absorption. Further, the electric field in neutron- and proton-irradiated pad detectors at an equivalent fluence of about 7 × 10^15^ $n_{\mathrm{eq}}/cm^{2}$ is investigated, where the space charge of the proton-irradiated devices appears inverted compared to the neutron-irradiated device.

## 1. Introduction

The Transient Current Technique (TCT) is a tool for the characterisation and investigation of detector technologies [1]. Excess charge carriers are generated via pulsed laser light inside the device under test (DUT), and the excess carrier’s drift is studied to obtain information about the DUT. TCT is widely used for the characterisation of silicon detectors that are used within high-energy physics experiments [2,3].

The change in pulse irradiance *I* along the propagation direction *z* is described by [4]

(1) $$\frac{dI(r,z,t)}{dz}=-\alpha I\left(r,z,t\right)-\beta_{2}I^{2}\left(r,z,t\right)-\sigma_{ex}NI\left(r,z,t\right)$$

with *r* being the distance to the beam axis. α and $\beta_{2}$ are the linear (single-photon, SPA) and non-linear (two-photon, TPA) absorption coefficients. $\sigma_{ex}$ and *N* are the cross-section for free carrier absorption and the number of free charge carriers. Conventional TCT uses red or near-infrared light to generate excess charge carriers in silicon. The number of charge carriers created at these wavelengths grows linearly with the light intensity. Compared to conventional TCT, the Two-Photon Absorption–Transient Current Technique (TPA-TCT) uses light in the quadratic absorption regime (here 1550 nm) to generate excess charge by two-photon absorption [5]. The generation of excess charge carriers by TPA depends quadratically on the light intensity, which is why focused laser light dominantly generates excess charge in a small volume (here about $60\;\mu m^{3}$) around the focal spot. Thus, TPA-TCT enables device characterisation with three-dimensional spatial resolution [6]. The technique was developed in the framework of the RD50 collaboration [7,8], and a table-top setup for the characterisation of silicon detectors was commissioned at CERN [9]. The development of radiation hard detectors for future detector technologies requires suitable characterisation techniques to obtain a profound understanding about the devices, before and after irradiation. The potential of the TPA-TCT as a characterisation tool has already been demonstrated in non-irradiated devices [10], but besides a few studies on irradiated devices [11,12], a systematic investigation of the TPA-TCT in irradiated devices for different particle types has so far not been reported. This paper is dedicated to such a systematic investigation of the influence of neutron, proton, and gamma irradiation of different fluences and doses on the TPA-TCT. The reported measurements were conducted and published within a broader framework in a PhD thesis [13].

## 2. Experimental Setup

**Figure 1:**
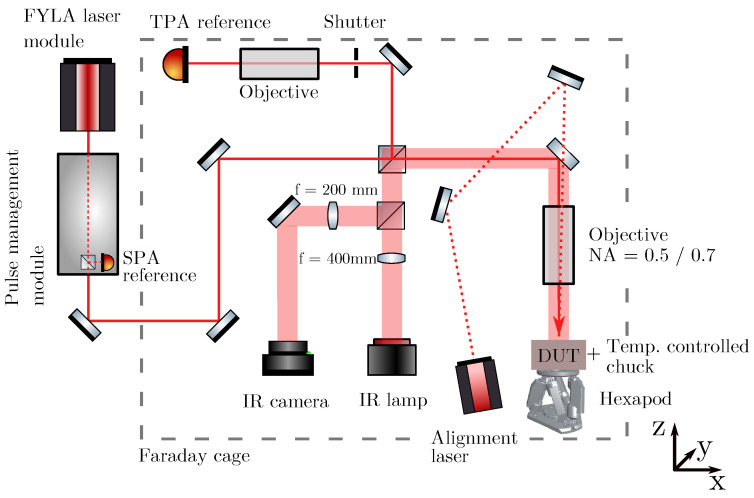
Sketch of the used table-top TPA-TCT setup.

For the present study, a table-top TPA-TCT setup is used, which is shown as a schematic in Figure 1.

The FYLA LFC1500X fibre laser module is used as a laser source [14]. The central wavelength is 1550 nm, the temporal pulse width is about 430 fs, the output pulse frequency is 8.2 MHz, and the pulse energy is 10 nJ at its output. Downstream of the output, the light is guided in a pulse management module, where an acousto-optic modulator regulates the pulse frequency and a neutral density filter adapts the pulse energy to given values. For the measurements presented here, a pulse frequency of 200 Hz is used, and the laser intensity is scanned between 40 pJ to 250 pJ (measured at the position of the DUT). The DUTs are cooled to −20 °C during the measurement, and the Faraday cage is continuously flushed with dry air to avoid freezing. The beam radius at the waist is $w_{0}=1.2\;\mu m$, and the Rayleigh length is $z_{R}=9.7\;\mu m$. In front of the objective, 50% of the light is coupled to a second arm by a beam splitter. The second arm is used for the energy reference, where a 300 μm thick p-type silicon pad detector serves as a reference for the charge generation by TPA.

The DUTs are glued with silver epoxy to a printed circuit board (PCB), which establishes the electrical connection. The PCB is mounted below the objective to a copper chuck that is thermally coupled to a Peltier element, where the hot side of the Peltier element is cooled by a HUBER chiller. In order to lower the leakage current of the DUTs, the active cooling to (−20.0±0.1) °C is performed, and the Faraday cage is continuously flushed with dry air. The illumination is applied from the top side into the centre of the opening window of the device. The copper chuck is positioned on a six-axes Newport HXP50-MECA stage, which allows high-precision movement and rotation along all three-dimensional axes. The rotation is used to level the DUT and ensure an orthogonal incidence of the laser. The setup includes an infrared (IR) microscope, with an IR lamp and IR camera. The IR microscope is used to live picture the DUT below the objective and to find regions of interest. Further information about the setup can be found in reference [5].

The data acquisition is handled by an Agilent DSO9254 oscilloscope, with a bandwidth of 2.5 GHz and a sampling rate of $20\;GSa/s\hat{=}50\;ps/pt$. To obtain induced current signals of a suitable amplitude, the 40 dB C2HV transimpedance amplifier from CIVIDEC [15] is used. It has a bandwidth from 10 kHZ to 2 GHz.

### Devices

Planar p-type pad silicon detectors are used to investigate the influence of irradiation on the TPA-TCT. Pad detectors are selected, as their simple device design allows to distinguish radiation-induced, technique-related, and device-related effects. The devices were manufactured by CiS [16] in the campaign CiS16 from FZ p-type bulk material with a pre-irradiation resistivity >10kΩcm. The thickness of the high-resistivity bulk, which can be depleted by applying a sufficiently high reverse bias voltage, is called active thickness. The active thickness and irradiation and annealing information of the devices is summarised in Table 1. The pad detectors have a circular opening in the top metal with a diameter of 1.2 mm, which is ideal for light-based characterisation techniques like the TPA-TCT.

Neutron irradiation was performed at the TRIGA reactor at the Jožef Stefan Institute [17], proton irradiation at the PS-IRRAD proton facility at CERN [18], and gamma irradiation at the Ruđer Bošković Institute [19]. The neutron irradiation in the TRIGA reactor has a broad energy spectrum which is converted by the facility operators into the 1 MeV equivalent fluence [20]. This is why $n/cm^{2}=n_{\mathrm{eq}}/cm^{2}$ within this work. The notation $n/cm^{2}$ is still used to distinguish the type of irradiation from the proton irradiation, where $p/cm^{2}$ states the absolute proton fluence. The PS-IRRAD proton facility uses 23 GeV protons for the irradiation, and the hardness factor of the facility is $\kappa_{23Ge\;V\;p}=0.62\pm 0.01$. The gamma irradiation was performed with a ^60^Co source. No hardness factor can be given for the gamma irradiation due to the completely different damage mechanism. Fluences and doses as well as the annealing states of the devices are included in Table 1.

## 3. Influence of Radiation Damage

Radiation damage does not only affect the device performance but can also influence the characterisation technique. The influence of radiation damage on the TPA-TCT is systematically studied using a series of irradiated pad detectors. Such a study is needed in order to distinguish technique-related effects from device-related effects, which is especially relevant with respect to segmented devices.

### 3.1. Correction of the Single-Photon Absorption Offset

Defects can introduce energy levels within the band gap of silicon, which lead to additional linear absorption [21]. This additional single-photon absorption contribution is called single-photon absorption (SPA) background or SPA offset. The SPA background is independent of the focal point’s position and appears as a constant offset in a charge collection scan. Three different methods to correct the SPA offset are available: correction by subtraction [12], correction by intensity [9], and correction by waveform subtraction [6]. The prior is the simplest method, where a constant is fitted to the offset and subtracted from the charge profile. It does not provide a correction on the waveform level, which means that it does not correct the influence of the SPA contribution towards the shape of the current transient. Due to its simplicity, it is useful to correct collected charge (CC) profiles, but it is not applicable to correct, e.g., the prompt current or the time over threshold (ToT). The intensity method requires the measurement to be recorded twice at different laser intensities. For the correction, the fact that the SPA and the TPA contribution scale differently with the laser intensity is exploited. The method was developed to cope with laser beam clipping, as it compares two measurements and thus corrects intrinsic clipping and reflection. Reference [9] contains more information about the method and derives the relevant formulas. The third and preferred method is the waveform subtraction method. A waveform is recorded with the focal spot above (i.e., outside of) the active volume. This waveform contains a negligible amount of TPA and is dominated by the SPA contribution. As the SPA contribution is independent on the focal spot’s position, this waveform is subtracted from all other waveforms to cancel out the SPA contribution. The method is only valid if the laser intensity is constant throughout the measurement, i.e., no laser beam clipping or reflection is present.

**Figure 2:**
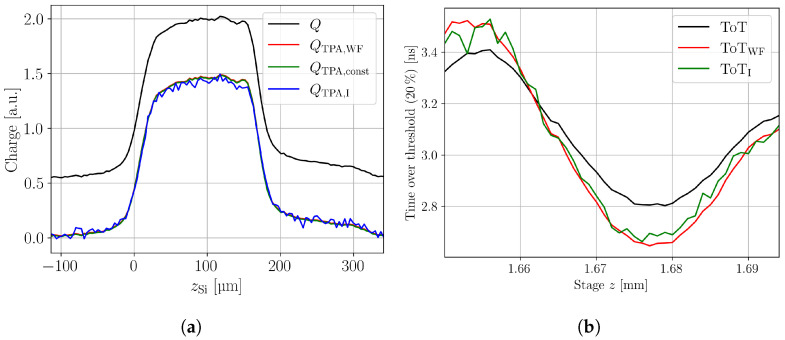
(**a**) Charge versus depth profile of an irradiated (3.32 × 10^14^ n/cm) 156 μm thick p-type planar pad detector. The bias voltage is 300 V. A comparison of the three different SPA correction methods is shown. The waveform subtraction method is indicated by the index WF, the subtraction method by the index const , and the correction by intensity method by the index I. The uncorrected data are given without index. (**b**) Time over threshold measurement in the same pad detector, including a comparison of the waveform subtraction and the intensity correction method.

Figure 2 shows a comparison between the three methods using a neutron-irradiated pad detector as an example. The correction of the CC profile is shown in (a) and the correction of the ToT profile in (b). Concerning the CC, most striking is the increased noise in the intensity correction method compared to the other methods. This is related to the need to take two measurements at different intensities: in this case, one measured at about 65 pJ and one measured at 320 pJ. The low intensity measurement has a lower signal-to-noise ratio (SNR), which propagates through the correction method and increases the noise. Such different intensities are needed for the correction method, because too close intensities lead to numerical instability. The subtraction method does not appear in Figure 2b, because it does not provide a correction on the waveform level and thus cannot be used to correct the ToT. The waveform subtraction method is the recommended method for SPA correction and used within this work.

### 3.2. Influence of Neutron and Proton Irradiation

**Figure 3:**
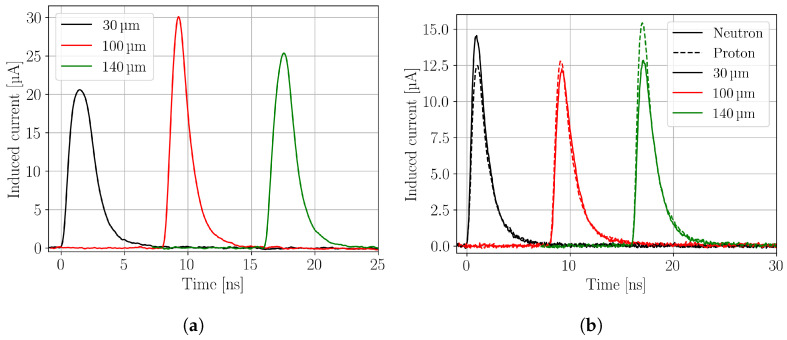
Current transients recorded in the non-irradiated 21-DS-79 (**a**), the neutron-irradiated 21-DS-102, and the proton-irradiated 21-DS-92 (**b**) CiS pad detector. The fluence of the neutron- and proton-irradiated device were 7.02 × 10^15^ $n/cm^{2}$ and 1.17 × 10^16^ $p/cm^{2}$, respectively. Positions in the legends refer to positions of the focal point where 0 μm corresponds to the top side and 156 μm to the back side of the sensor. The measurements were performed at −20 °C and 0% relative humidity. The beam parameters were $w_{0}=1.2\;\mu m$ and $z_{R}=9.7\;\mu m$, and a pulse energy of 200 pJ was used. The laser frequency was 200 Hz, and the average of 256 single acquisitions was recorded. The bias voltage was 300 V. The signals are shifted on the time axis for better readability.

Measured current transients of an unirradiated, a neutron-, and a proton-irradiated sample are shown in Figure 3 for different depths (30 μm, 100 μm and 140 μm) of the focal point in the active volume. The signals are shifted on the time axis for better readability.

**Figure 4:**
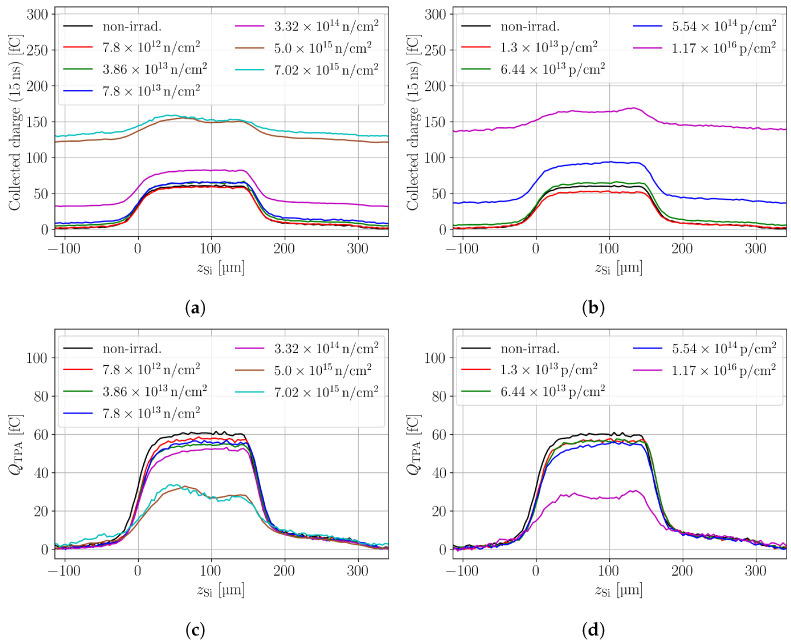
In-depth scans of the charge collection in pad detectors for neutron- (**a**) and proton- (**b**) irradiated samples. Figure (**c**,**d**) show the SPA corrected in-depth scans for neutrons and protons, respectively. The bias voltage is 300 V.

CC profiles of the neutron- and proton-irradiated samples are shown in Figure 4a,b, respectively. It can be seen that both lead to an SPA offset that increases with the fluence. Figure 4c,d show the SPA corrected in-depth scans, where the decreasing CC is evident. Further, it can be seen that increasing fluences lead to inhomogeneous CC profiles along the device depth. The inhomogeneity is linked to inhomogeneous electric fields and charge-carrier-dependent trapping. Depending on the excess charge deposition depth, electrons or holes need to drift a longer distance and face different low field regions, which is why their probability of trapping changes with the deposition depth.

**Figure 5:**
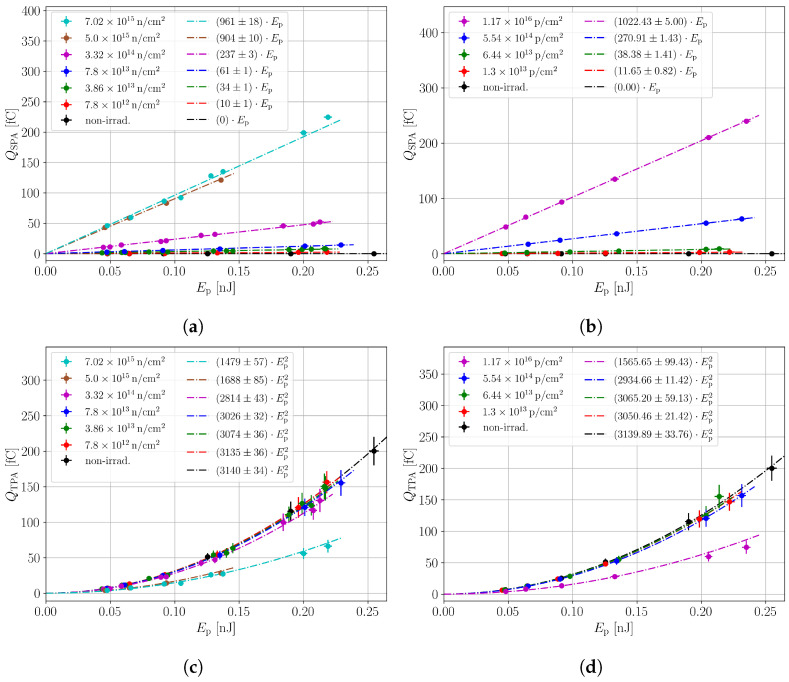
Charge collected by SPA in neutron- (**a**) and proton- (**b**) irradiated 156 μm thick pad sensors. Charge collected by TPA for the same neutron- (**c**) and proton- (**d**) irradiated devices. The TPA charge is extracted as the mean of the collected charge between the FWHM of the in-depth scans. The bias voltage for all scans is 300 V.

In-depth scans were performed at various bias voltages and laser intensities in order to investigate the linear and quadratic dependence of the SPA and the TPA contribution, respectively. The SPA contribution to the collected charge $Q_{\mathrm{SPA}}$ is extracted as the mean CC in a range between $-265\;\mu m\leq z_{\mathrm{Si}}\leq -130\;\mu m$ in front of the device. In this region, charge generation by TPA can be neglected. The TPA contribution to the collected charge $Q_{\mathrm{TPA}}$ is extracted from the SPA corrected CC profiles as the mean between the full width at half maximum (FWHM) of the CC profile. It should be noted that the analysis procedure of $Q_{\mathrm{TPA}}$ can be performed at a given depth of the device to investigate the CC along the device depth. Here, the average over the device depth is used to ease the analysis procedure. Figure 5 shows the CC from SPA (a) and TPA (c) against the laser intensity. In accordance with the expectations, the SPA contribution scales linearly and the TPA contribution quadratically with the laser intensity. The standard deviation of the averaged SPA and TPA charge yields the corresponding errors on the CC.

The analogous analysis was performed for the proton-irradiated samples. Figure 5b,d show the CC from SPA (b) and TPA (d) against the laser intensity. It can be seen that the proton-irradiated samples show a similar behaviour to the neutron-irradiated samples. A dedicated comparison between the neutron- and proton-irradiated samples is performed in Section 3.4.

**Figure 6:**
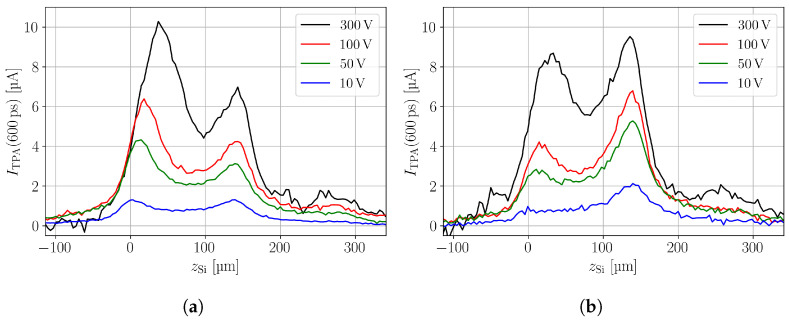
Prompt current at $t_{\mathrm{pc}}=600\;ps$ for different bias voltages measured for (**a**) a neutron fluence of 7.02 × 10^15^ $n/cm^{2}$ and (**b**) a proton fluence of 1.17 × 10^16^ $p/cm^{2}$. The equivalent fluences are comparable. The double junction is clearly visible in both plots. The proton-irradiated sample shows an electric field that grows from, and is stronger at, the back electrode. This effect is often called type inversion .

The prompt current (PC), i.e., the current measured at $t_{\mathrm{pc}}=600\;ps$ after start of transient, of the highest neutron- and proton-irradiated sample is shown in Figure 6a,b, respectively. The scans were performed at different bias voltages to emphasise the evolution of the electric field. Both samples show a clear double junction effect [22], as the electric field has maxima at both device boundaries. Further, the direction of electric field evolution is opposite for neutron- and proton-irradiated devices at the given fluence. While the electric field grows from the top (n^+^ electrode) towards the back side (p^+^ electrode) in the neutron-irradiated device, the direction of electric field evolution is opposite in the proton-irradiated device. The evolution of the electric field from the top towards the back is the typical direction of electric field in a p-type device, while the opposite is the case for n-type devices for which the electric field growth from the (p^+^ electrode). Thus, the space charge of the proton-irradiated device appears sign inverted compared to the neutron-irradiated device, which means that in the proton-irradiated sensor, a higher fraction of the bulk has obtained positive space charge as compared to the neutron-irradiated sensor. This is particularly interesting because space charge sign inversion (SCSI) in a p-type FZ detector has so far not been reported. However, the situation is complex because of the double junction, which makes the expression of SCSI somewhat ambiguous. The bulk is not fully transitioned from negative to positive space charge as this expression is stating but contains regions of negative and regions of positive space charge. For the neutron-irradiated devices, the negative space charge at the n^+^ electrode is higher than the positive space charge at the p^+^ electrode, while the ratio is inverted in the proton-irradiated devices.

For pad detectors, the found proton-irradiation-induced SCSI is not critical, because the weighting field is constant so that excess charge independent of its position contributes equally to the current transient. However, this effect could be significant to p-type strip or pixel detectors. Excess charge in the vicinity of the readout strip has the major contribution of the current transient, which is why the highest electric field is desired to be in that region [23]. Thus, the charge collection efficiency (CCE) of proton-irradiated p-type strip detectors might be decreased compared to neutron-irradiated devices at high equivalent fluences in segmented sensors.

Additionally, from the prompt current profile in Figure 6, it can be seen that the concept of depletion depth in dependence of bias voltage as defined for a pristine detector is not a useful concept any more. Already at the lowest shown bias voltage, an electric field is present across the full DUT’s active volume, and thus, full depletion in that sense is already reached, even though the CC will still increase with increasing bias voltage. Hence, to define the operational voltage of an irradiated detector, the CC is a better indicator. The operational voltage could be found as the voltage where the CC reaches a given threshold. This threshold needs to comply with the specifications of the intended use case.

### 3.3. Influence of Gamma Irradiation

**Figure 7:**
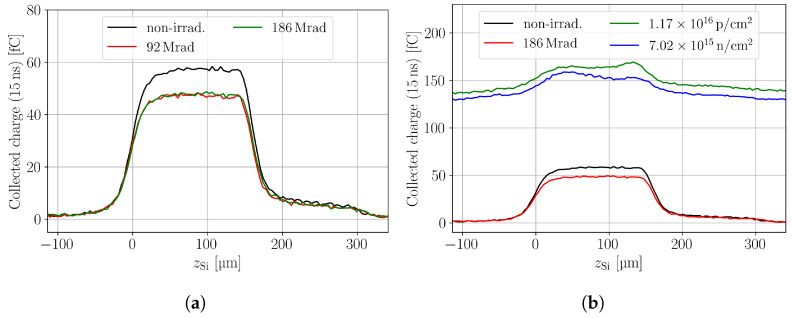
(**a**) In-depth scans of the charge collection in pad detectors. Gamma-irradiated samples and a non-irradiated sample are shown. (**b**) Comparison between the in-depth scans of a non-irradiated, a neutron-, a proton-, and a gamma-irradiated pad sensor. The bias voltage in all scans is 300 V.

The gamma irradiation was performed with a ^60^Co source at IRB in Zagreb [19]. Two pad sensors irradiated to 92.4 Mrad and 186.1 Mrad are studied. In-depth CC scans are shown in Figure 7a, where a non-irradiated device is shown for comparison. It is observed that the gamma irradiation, in contrast to neutron and proton irradiation, does not introduce an SPA background. A comparison between the CC in neutron-, proton-, and gamma-irradiated devices can be seen in Figure 7b. The gamma-irradiated samples show a decrease in CC with respect to the non-irradiated device, which indicates that trapping is present. At both doses, a comparable amount of charge is collected, which is confusing as it indicates a saturation in charge loss at these doses. Such saturation is not expected from the bulk leakage current measurements of the two devices which differed by a factor of about two. Also data on gamma-irradiated p-type sensors in the literature do not show a saturation effect for damage parameters like leakage current and depletion voltage in the dose range studied here [24]. Irrespective of this unresolved observation, it can be stated that the charge loss is constant throughout the two devices, contrary to the neutron- and proton-irradiated devices. Hence, the trapping is equal for all excess charge deposition positions, which suggests that the trapping cross-section of electrons and holes is approximately the same in the gamma-irradiated samples.

### 3.4. Comparison of Neutron, Proton, and Gamma Irradiation

As presented above, neutron, proton, and gamma irradiation have different influences on TPA-TCT measurements. The most striking difference between hadron- and gamma-irradiated samples is the SPA background found in the prior, as shown in Figure 7b. In the following, it will be argued that the SPA offset is related to cluster defects. Gammas from a ^60^Co source produce defects via Compton electrons, which do not have enough energy to create defect clusters but only point defects [25]. This is in contrast to hadron irradiation that creates both cluster and point defects. The concentrations of point defects introduced by neutron and gamma irradiation can be calculated using introduction rates from [26]. The most common point defects introduced by neutron and ^60^Co-gamma irradiation in high-resistivity silicon sensors are $\mathrm{VO},C_{i}C_{s}$, and $C_{i}O_{i}$. Neutron irradiation introduces both defects approximately proportional to the fluence. After a neutron fluence of 3.9 × 10^13^ $n/cm^{2}$, SPA offset is clearly visible, which corresponds to a concentration in the order of $\approx 4\times 10^{13}\;cm^{2}$ for the $\mathrm{VO}+C_{i}C_{s}$ and $C_{i}O_{i}$ defects. On the other hand, gamma irradiation introduces, at the highest investigated dose, about $\approx 1\times 10^{14}\;cm^{2}$ of these defects, and thus, a comparable or even higher concentration of those point defects is present. As the defect concentrations of the neutron and gamma irradiation are comparable, while the SPA offset is absent in the gamma-irradiated devices, it is concluded that the SPA offset is not caused by the discussed point defects. Compared to the $\mathrm{VO}+C_{i}C_{s}$ and $C_{i}O_{i}$ defects, other point defects and the $V_{2}$ defect are present in much smaller concentration and thus assumed to be less significant in the gamma-irradiated devices. This gives indication that the linear light absorption found in neutron- and proton-irradiated devices originates from cluster and/or $V_{2}$ defects that are not or only in very low concentration introduced by gamma irradiation. In [21] linear absorption of 1550 nm light in neutron-irradiated silicon is linked to the electrically neutral divacancy $V_{2}^{0}$ defect state, which is introduced by neutron irradiation within cluster defects [27]. This is in agreement with the hypothesis that the SPA background originates from cluster defects, which explains the absence of the SPA background for gamma-irradiated devices.

**Figure 8:**
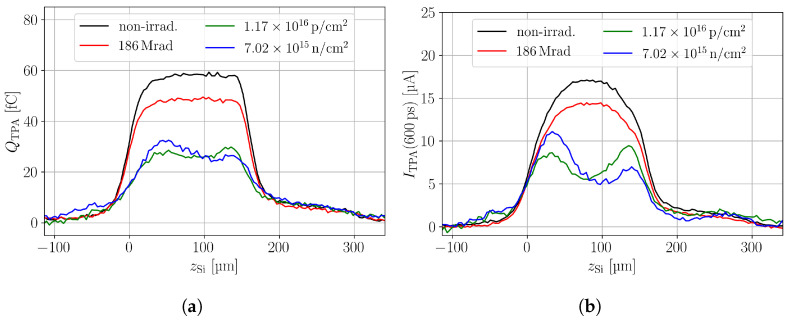
(**a**) In-depth scans of the charge collection in pad detectors. A non-irradiated sample is shown as well as a neutron-, a proton-, and a gamma-irradiated sample. (**b**) Same in-depth scans, but the SPA offset is corrected by the waveform subtraction method. The bias voltage is 300 V.

Figure 8 shows a comparison between the different irradiations for the highest fluence/dose available, where the SPA corrected CC (a) and PC (b) are shown. It can be seen that independent of the particle type, a decrease in the CC is present. Only the neutron- and proton-irradiated samples show variation along the device depth. Further, all particle types decrease the PC, i.e., the electric field in the device. Neutron and proton irradiation at the presented fluences leads to a double junction in the DUT, which is discussed in more detail in Section 3.2. Both the non-irradiated and the gamma-irradiated sample show a roundish-shaped PC profile, which does not agree with the expected linear behaviour in a pad detector. It is found that the roundish shape is an effect of CC during the PC time $t_{\mathrm{pc}}$. Smaller $t_{\mathrm{pc}}$ or lower bias voltages decrease the roundish shape and reveal the expected linear electric field shape throughout the device depth [13]. The effect is, here, especially visible as the charge is collected quickly at the used bias voltage that corresponds to about 2 V/μm. The PC time of $t_{\mathrm{pc}}=600\;ps$ is used for a sufficient SNR that allows an easy qualitative comparison among all samples.

**Figure 9:**
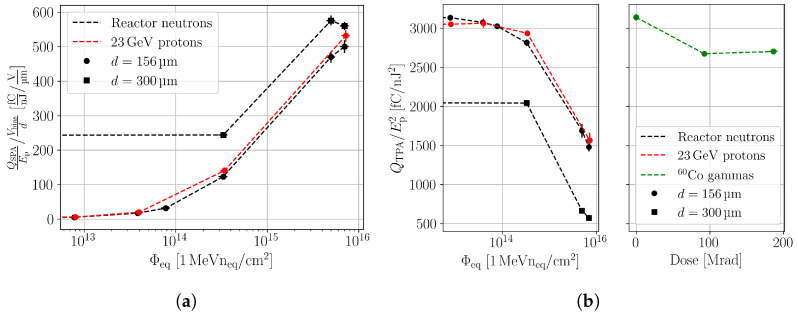
(**a**) SPA charge collection normalised with the average electric field versus the equivalent fluence for neutron- and proton-irradiated FZ p-type pad detector. (**b**) TPA charge collection versus the equivalent fluence and dose for the same DUT.

Figure 9 shows the normalised SPA (a) and TPA (b) contribution as a function of the equivalent fluence and dose. The SPA and TPA parameters are extracted from in-depth scans with the analysis procedure described in Section 3.2. To account for the device thickness dependence of the SPA, the SPA contribution is normalised with the bias voltage over the device thickness. Thicker devices collect more SPA charge due to the higher charge generation in the increased active thickness, and they collect less TPA-generated charge due to the longer drift times, as more charge carriers are trapped. It can be seen that the SPA and TPA contribution of neutron- and proton-irradiated samples scale similarly with the equivalent fluence, which indicates that these quantities scale with the NIEL. The scaling with the NIEL supports again the hypothesis that the SPA background originates from cluster damage.

**Figure 10:**
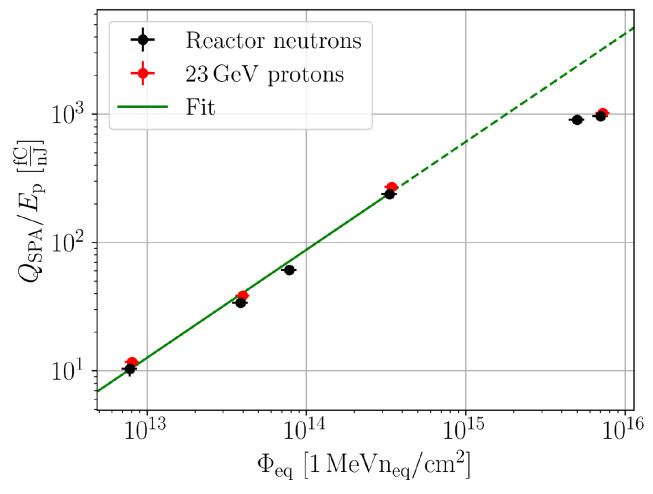
Charge collected by SPA normalised with the pulse energy for proton and neutron irradiation in 156 μm thick pad detectors, biased to 300 V. The fit function is of the form $C\;\cdot \;\Phi_{\mathrm{eq}}^{a}$, with $C=14.3\times 10^{-11}\;cm^{2a}fC/nJ$ and a=0.84. The highest fluences are excluded from the fit.

To parametrise the SPA background against the equivalent fluence, it is plotted in a log-log diagram, as shown in Figure 10. The SPA contribution follows for fluences $<1\times 10^{15}\;n_{\mathrm{eq}}/cm^{2}$ a function $C\;\cdot \;\Phi_{\mathrm{eq}}^{m}$. Higher fluences deviate from this behaviour, and it was measured that increasing bias voltages reduced the deviations, which is not shown in detail within this manuscript. This bias dependence indicates that the deviations are related to charge trapping that becomes apparent at the highest fluences.

### 3.5. Beam Depletion Due to SPA

The additional SPA contribution in irradiated devices leads to additional beam depletion, which in turn potentially leads to a decreasing TPA charge generation along the device depth. To estimate the beam depletion due to linear absorption, an effective absorption coefficient can be defined from the collected SPA charge generation:

(2) $$\alpha_{\mathrm{eff}}=\frac{-1}{d}\ln\left(1-Q_{\mathrm{SPA}}\frac{\hbar \omega }{eE_{p}}\right)\;.$$

The quotient $Q_{\mathrm{SPA}}/E_{p}$ is obtained from the fitting of the SPA contribution (see Figure 5a,b). The absorption coefficient is called effective, because only absorption that generates charge that is collected is considered. When there is absorption that does not lead to excess charge carriers, or if the excess charge carriers are trapped and not measured, they are not accounted in the effective absorption coefficient. In general, the inequality $Q_{\mathrm{SPA},\mathrm{coll}}\leq Q_{\mathrm{SPA},\;\mathrm{gen}}$ applies. Equality is reached in the absence of charge loss. When defects that absorb light but do not generate excess charge are present, or charge loss is present, the inequality yields the following:

(3) $$\alpha_{\mathrm{eff}}\leq \alpha_{\mathrm{irrad}}\;,$$

with $\alpha_{\mathrm{irrad}}$ being the linear absorption coefficient due to irradiation, which is defined as follows:

(4) $$\alpha_{\mathrm{irrad}}=\alpha \left(\Phi_{\mathrm{eq}}\right)-\alpha \left(0\right)\;.$$

**Figure 11:**
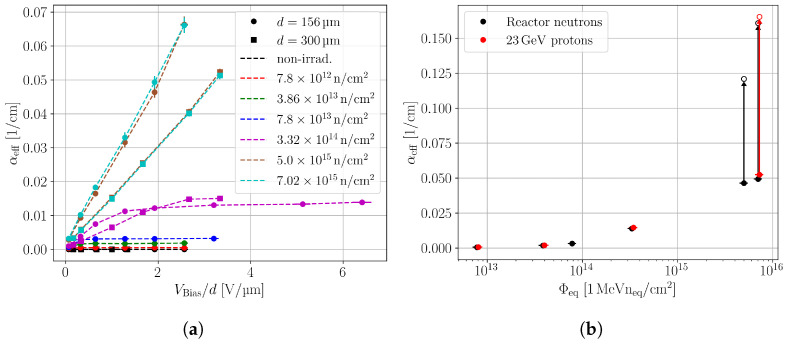
(**a**) Effective linear absorption coefficient of neutron-irradiated pad detectors versus the bias voltage, which is normalised with the device thickness. (**b**) Effective linear absorption coefficient for 156 μm thick neutron- and proton-irradiated pad detectors versus the equivalent fluence. The value for the maximum bias voltage is used. The absorption coefficient does not saturate for the highest fluences, and the highest applied bias voltage is different for the proton- and neutron-irradiated devices. In order to show comparable $\alpha_{\mathrm{eff}}$ for the highest fluences, a bias voltage of 300 V is selected. The arrows are used to guide the eye towards the empty markers. These show the $\alpha_{\mathrm{eff}}$ calculated from the interpolated $Q_{\mathrm{SPA}}\left(\Phi_{\mathrm{eq}}\right)$ of Figure 10.

Figure 11a shows the effective linear absorption coefficient versus the bias voltage for neutron-irradiated pad sensors with different thicknesses. $\alpha_{\mathrm{eff}}$ increases with the bias voltage and saturates within the investigated voltage range for fluences up to 3.3 × 10^14^ $n/cm^{2}$. The maximum absorption coefficient for these fluences is below $0.02\;cm^{-1}$, which leads to a beam depletion below 0.1% in a 300 μm thick silicon detector. Such a low-intensity loss can be neglected. Thicker devices yield lower $\alpha_{\mathrm{eff}}$, because the charge loss is more pronounced, which increases the inequality of Equation (3). Higher fluences do not reach saturation within the used bias voltage range, because the charge loss does not saturate up to the maximum bias voltage. Figure 11b shows the effective absorption coefficient for neutron- and proton-irradiated pad detectors versus the equivalent fluence. As the SPA charge collection does not saturate for the highest fluences, only a lower limit for $\alpha_{\mathrm{eff}}$ is stated. From the measured data, it is ensured that the beam is not depleted by the SPA absorption for fluences up to at least 3.32 × 10^14^ $n/cm^{2}$. The effective absorption coefficient does not saturate for higher fluences, which hinders the investigation of the absolute beam depletion. Reference [21] measures $\alpha_{\mathrm{irrad}}<1\;cm^{-1}$ for a fluence of $10^{16}\;n/cm^{2}$ in non-biased detector grade silicon, which corresponds to less than 3% absorption in a 300 μm thick device. In conclusion, beam depletion in irradiated devices due to SPA for fluences up to at least $10^{16}\;n/cm^{2}$ is assumed to be negligible.

### 3.6. Influence on the Two-Photon Absorption Coefficient

In the above presented measurements, it is observed that irradiation decreases the charge collection. However, the reliability of the collected data is not ensured, because the charge generation mechanism of TPA might be impacted by the irradiation, meaning that the absorption coefficient $\beta_{2}$ might be a function of fluence. To investigate the fluence dependence of $\beta_{2}$, a comparative CC study was performed using a ^90^Sr setup. Charge sensitive amplifiers are used to increase the SNR. The 156 μm thick, neutron-irradiated subset is used for this study, and the non-irradiated detector from the same wafer serves as a reference.

**Figure 12:**
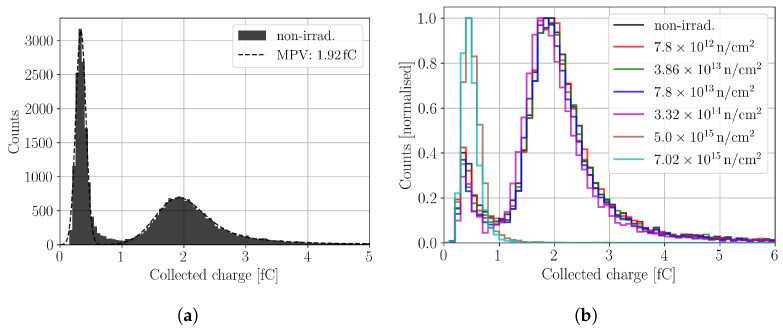
(**a**) Charge collection of a non-irradiated 156 μm thick p-type pad sensor measured in a ^90^Sr setup. (**b**) Charge collection in neutron-irradiated pad sensors for different fluences. The histogram is normalised to the maximum amount of counts to ease the comparison. The bias voltage in all measurements is 300 V.

An example for the CC measurement in a non-irradiated device in shown in Figure 12a. It can be seen that the CC resembles the expected Landau–Gauss distribution, and an additional noise peak for charge <1 fC is measured. The main part of the CC distribution is fitted with a Landau–Gauss distribution, and the noise peak is fitted by a Gaussian distribution [28]. The used fitting functions yield a satisfactory agreement with the data and allow to extract the most probable value (MPV) from the distribution, which corresponds to the generated charge of a minimum ionising particle (MIP). The MPV (1.92 fC) needs to be corrected with the mean of the noise floor (0.33 fC) to obtain the MIP charge. For a non-irradiated detector, the MPV is found as 1.59 fC≈9924 electron–hole pairs, which is in good agreement with expectations from the literature [29].

Figure 12b shows the evolution of the CC with increasing neutron irradiation. To suppress noise as efficiently as possible, the distributions are filtered by the following criteria: the peaking time of the DUT needs to agree within a time frame of ±50 ns around the peaking time of the reference. When the CC of the non-irradiated device in Figure 12b is compared to Figure 12a, it is evident that the used criteria suppresses noise efficiently. Even after the applied criteria, some noise remains, because the noise is randomly distributed in time. It is observed that the CC decreases with fluence due to the increasing charge trapping. At a certain fluence, the signal and noise distribution begin to overlap, which hinders the extraction of the MPV. Here, for fluences $\geq 5\times 10^{15}\;n/cm^{2}$, the extraction of the MPV is no longer possible.

**Figure 13:**
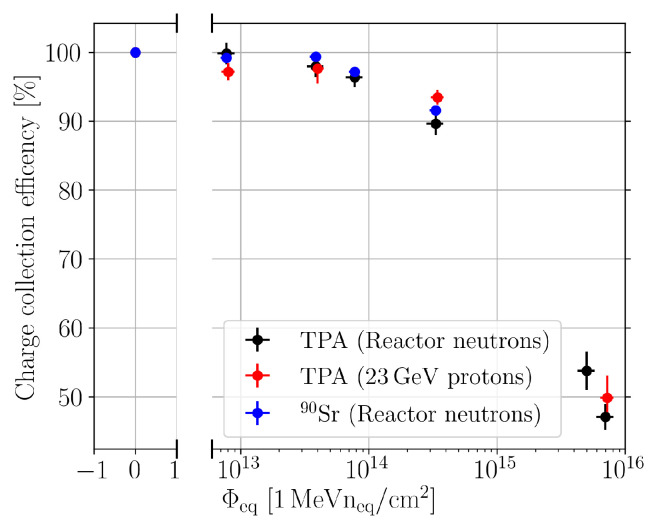
Charge collection efficiency for pad sensors, measured with the TPA-TCT and a ^90^Sr setup, against the equivalent fluence. The type of irradiation is mentioned in the legend.

Figure 13 shows charge collection efficiency (CCE) against the equivalent fluence measured with the TPA-TCT setup and the ^90^Sr setup. The CCE is defined as the percentage of charge that is collected in comparison to the non-irradiated device. For the ^90^Sr setup, this means the normalisation of the MPV with the MPV of the non-irradiated device, and for the TPA-TCT, the fitting parameters for $Q_{\mathrm{TPA}}$ described in Section 3.2 are normalised with the fitting parameters from the non-irradiated device. The data from the proton-irradiated devices are included even though they were only measured with the TPA-TCT. It can be seen that both techniques yield, within the measurement uncertainty, a compatible CCE. The compatibility of both techniques indicates that the charge generation mechanism of TPA and the β particle of the ^90^Sr source scales similar with fluence up to at least 3.32 × 10^14^ $n/cm^{2}$. As the excess charge generation by the two process is very different from the ionisation process of a β particle, the similarity of the CCE measurements strongly hints that the charge generation mechanism of TPA, i.e., the absorption coefficient $\beta_{2}$, is constant with irradiation $\frac{d}{d\Phi }\beta_{2}\left(\Phi \right)=0$ up to a fluence of at least 3.32 × 10^14^ $n/cm^{2}$. Contrary to $\beta_{2}$, the SPA coefficient α is fluence-dependent (see Section 3.1 and [21]).

Besides the investigation of $\beta_{2}$, it can be seen that the TPA-TCT provides CCE values even for fluences $\geq 5\times 10^{15}\;n/cm^{2}$ that are not accessible with the used ^90^Sr setup. This has two reasons: first, the intensity of the laser can be increased to much more than one MIP equivalent of charge, and second, averages for multiple acquisitions are available, as the charge generation of the laser is reproducible pulse-per-pulse. Both reasons significantly increase the SNR. In contrast, the ^90^Sr setup relies on single acquisition, because the charge generation is a stochastic process, and the events are uncorrelated. Further, the setup triggers on the coincidence of the two reference detectors, and in order to mitigate influences on the DUT’s signal acquisition, the DUT is not included in the trigger. Hence, it is not ensured that the DUT records a particle hit for every acquisition, which leads to the noise floor. In conclusion, it was demonstrated that the TPA-TCT allows for the performing of CCE measurements similar to a ^90^Sr setup. The TPA-TCT offers an extended measurement range. Moreover, the TPA-TCT allows us to study the CCE against the device depth, which was not shown here, because the aim of these measurements was to investigate the effects of radiation damage on $\beta_{2}$, wherefore a study of a depth-dependent CCE was not presented, and the mean over the full device depth was used.

### 3.7. Refractive Index

**Figure 14:**
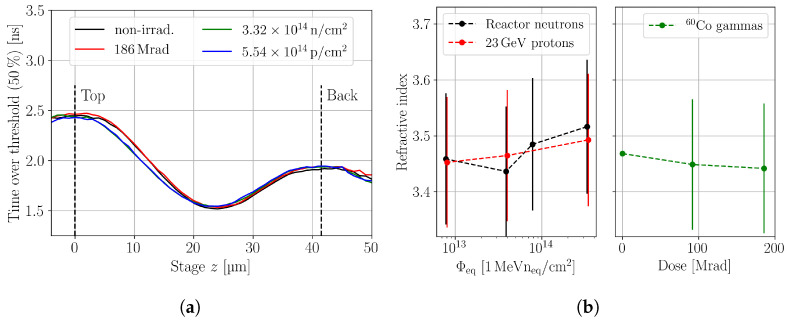
(**a**) Time over threshold profiles of the non-irradiated and the irradiated pad detectors. The highest fluences, while avoiding the double junction effect, are used to allow the comparison. The refraction from the air–silicon interface is not corrected in the *z*-axis. The location of the top and back interface is extracted from the non-irradiated device, and they are indicated by the dashed lines. (**b**) Refractive index extracted from the in-depth scans of various irradiated pad detectors. The nominal refractive index n(250 K)=3.4681±0.0002 is taken from [31]. All scans are performed with a bias voltage of 300 V.

Irradiation potentially changes the refractive index of silicon, as was observed for the bombardment with heavy ions [30]. A changing refractive index influences the reflectance of the material and, thus, the amount of generated charge. In this section, the refractive index is studied indirectly from TPA-TCT in-depth scans by the exploitation of the refraction at the top air–silicon interface. Refraction is described by [9]:

(5) $$z_{\mathrm{Si}}=z\;\cdot \;\sqrt{\frac{\pi n^{3}z_{R}}{\pi nz_{R}-\lambda n^{2}+\lambda }}\;,$$

with the refractive index *n*, the Rayleigh length $z_{R}$, and the wavelength λ. The equation can be rearranged to the squared refractive index

(6) $$n^{2}=-\frac{\gamma -s^{2}}{2}+\sqrt{\frac{{\left(\gamma -s^{2}\right)}^{2}}{4}+\gamma }\;,$$

with $\gamma =\left(\lambda s^{2}\right)/\left(\pi z_{R}\right)$ and $s=z_{\mathrm{Si}}/z$. Therefore, a study of the scaling allows for the drawing of conclusions on the refractive index. If irradiation changes the refractive index, the scaling factor would depend on the fluence and dose, so that the boundary interfaces of the devices would appear at different positions in the uncorrected coordinate system. As all devices are from the same wafer, the assumption of the same thicknesses is reasonable. The position of the boundaries is extracted from the in-depth scan. The back side surface is found from symmetries in the ToT profile, wherefore such plots are well suited to qualitatively compare the devices thicknesses. Figure 14a shows ToT profiles in uncorrected *z*-coordinates, where the positions on the top and back side surface extracted from the non-irradiated device are indicated. It is visualised that all devices, independent of their irradiation, have the interfaces at about the same position, which indicates that the refractive index is expected to be reasonably independent of irradiation. Aside from the boundary positions, the hadron-irradiated devices show differences in the ToT at the top side and only slight differences at the back side. The difference at the top side is related to hole trapping, which is more severe than the electron trapping; thus, a less significant difference is present at the back side.

A quantitative study of the refractive index is performed using the scaling factor, which is found by comparing the extracted device thicknesses in stage coordinates, i.e., uncorrected coordinates. The boundaries are extracted from fits towards the SPA corrected charge profile of all devices individually. The scaling factor is found by comparison with the non-irradiated device, and the refractive index is then calculated from equation (6) This procedure is performed for all devices that do not show the double junction effect, because the double junction is found to dominate the ToT profile, which hinders the extraction of the device’s boundaries. The results are summarised in Figure 14b. No trend with the equivalent fluence is observed, and all measured values are compatible with the nominal value of the non-irradiated device. The error bars are quite large, because the used method is only indirect, and many measurement uncertainties propagate to yield the presented errors. In conclusion, it is found that if irradiation changes the refractive index, the change due to equivalent fluences up to 3.32 × 10^14^ $cm^{-2}$ and doses up to 186 Mrad is <5.5% to its nominal value. Therefore, a fluence-independent refractive index is a reasonable assumption for the investigated fluence range.

## 4. Conclusions

Neutron-, proton-, and gamma-irradiated pad detectors were systematically studied using the TPA-TCT. A main finding was the absence of a single-photon absorption (SPA) background for gamma-irradiated devices, contrary to irradiation with neutrons or protons. For the latter, it was found that the SPA background scales with the equivalent fluence, which indicates that it is related to bulk damage and in accordance with the non-ionising energy loss (NIEL) hypothesis. The functional relation for the SPA-induced charge was measured to be $C=14.3\times 10^{-11}cm^{2\;\cdot \;0.84}fC/nJ\;\cdot \;\Phi_{\mathrm{eq}}^{0.84}$ for 156 μm thick sensors. The absence of the SPA background in gamma-irradiated devices and the scaling of the SPA background with NIEL indicate that the occurrence of SPA is related to cluster damage. Differences in the prompt current profile between neutron- and proton-irradiated devices were observed for $\approx 7\times 10^{15}\;n_{\mathrm{eq}}/cm^{2}$. Both devices showed a double junction, and the proton-irradiated device appeared *type-inverted* compared to the neutron-irradiated device, meaning that the electric field starts to grow from the back electrode with rising voltage. This proton-irradiation-related inversion could be relevant to p-type strip detectors as it potentially leads to a decreased charge collection efficiency (CCE) compared to similar neutron-irradiated strip detectors.

It was measured that the additional SPA contribution does not lead to a meaningful beam depletion in silicon devices up to fluences of at least 3.3 × 10^14^ $n_{\mathrm{eq}}/cm^{2}$, which is important in order to obtain comparable charge generation along the device depth of irradiated devices. Further, it was verified that the refractive index and the absorption coefficient $\beta_{2}$ agree with their nominal values for fluences up to at least 3.3 × 10^14^ $n_{\mathrm{eq}}/cm^{2}$. In general, it was demonstrated that the TPA-TCT produces meaningful results for irradiated devices and is a valuable addition to the present set of available characterisation methods.

## Figures and Tables

**Table 1:** Details of the used pad sensors. All devices are fabricated by CiS in the CiS16 campaign and have a FZ p-type bulk with a pre-irradiation resistivity >10kΩcm. The proton fluences are given in absolute values, while the neutron fluences represent the 1 MeV neutron equivalent fluences.

Name	Active	Fluence/	Annealing
	Thickness [μm]	Dose	
Pristine			
21-DS-79	156 μm	–	–
25-DS-66	300 μm		
Neutron [$n/cm^{2}$]			
21-DS-78	156 μm	$7.80\times 10^{12}$	10min at 60 °C and 6600min at 20 °C
21-DS-84		$3.86\times 10^{13}$	
21-DS-98		$7.80\times 10^{13}$	
21-DS-99		$3.32\times 10^{14}$	
21-DS-101		$5.00\times 10^{15}$	
21-DS-102		$7.02\times 10^{15}$	
25-DS-104	300 μm	$3.32\times 10^{14}$	
25-DS-87		$5.00\times 10^{15}$	
25-DS-88		$7.02\times 10^{15}$	
Proton [$p/cm^{2}$]			
21-DS-97	156 μm	$1.3\times 10^{13}$	10min at 60 °C and 6600min at 20 °C
21-DS-96		$6.44\times 10^{13}$	
21-DS-94		$5.54\times 10^{14}$	
21-DS-92		$1.17\times 10^{16}$	
Gamma [Mrad]			
19-DS-97	156 μm	92.4	-
19-DS-99		186.1	

## Data Availability

The data presented in this study are available on request from the corresponding author. The data are not publicly available due to the complexity of the data and to maintain an overview on usage.

## Abbreviations

The following abbreviations are used in this manuscript:

CC	Collected charge
CCE	Charge collection efficiency
DUT	Device under test
FWHM	Full width at half maximum
MIP	Minimum ionising particle
MPV	Most probable value
PCB	Printed circuit board
SNR	Signal-to-noise ratio
SPA	Single Photon Absorption
TCT	Transient Current Technique
ToT	Time over threshold
TPA	Two-Photon Absorption
